# The Orthopaedic Trauma Association's international relations: an expanding initiative

**DOI:** 10.1097/OI9.0000000000000190

**Published:** 2022-04-18

**Authors:** Hans-Christoph Pape

**Affiliations:** Universitätsspital Zürich, Zürich, Switzerland.

The **Orthopaedic Trauma Association (OTA)** has come a long way from being a North American Association for fracture care to becoming one of the most respected international organizations for gathering new knowledge regarding musculoskeletal trauma, developing injury classifications, coordinating interdisciplinary courses—all while adhering to its original mission of being an educational resource for residents, fellows, attending physicians, physician assistants, and nurses.

Several important steps have been undertaken to amplify its radiance as an educational organization. Internally, the addition of courses for residents and physician assistants has broadened the spectrum of educational offerings for trainees and allied health providers.

Externally, the addition of regular *Guest Nation Programs* at the OTA Annual Meeting and the formation of an *Intl.Committee* has attracted a growing body of international members and guests.

Moreover, the foundation of a *Humanitarian Committee* and the active participation ofOTAcore members in various countries has been well-received and has led to many new collaborations in education and research. Relationships have been formed and strengthened with other societies, such as the OTA's participation in the *World Trauma Congress* in association with DKOU in Frankfurt in 2014. Moreover, the importance of interdisciplinary fracturemanagement has been reinforced by active participation of OTA members during the *Polytrauma Courses* throughout the world (www.polytraumacourse.com).

One of the most important milestones has certainly been the initiation of **IOTA** in 2017 with the key figure of Dr Ted Miclau. What started with a single meeting soon became a regular exchange between international surgeons. Although interrupted by the pandemic, the first OTAI International meeting is planned to take place in Amsterdam in December 2022. For the first time, OTA will also play an active role at the upcoming ESTES (*European Society of Trauma and Emergency Surgery*) meeting in Oslo in 2022.

**Figure 1:**
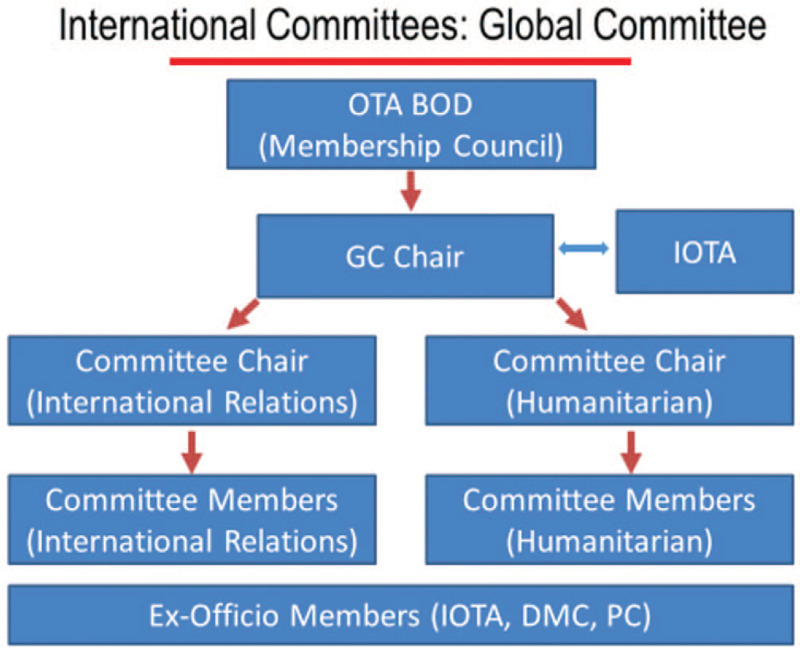
Humanitarian committee, Intl. Committee, Global Relations committee.

**Figure 2:**
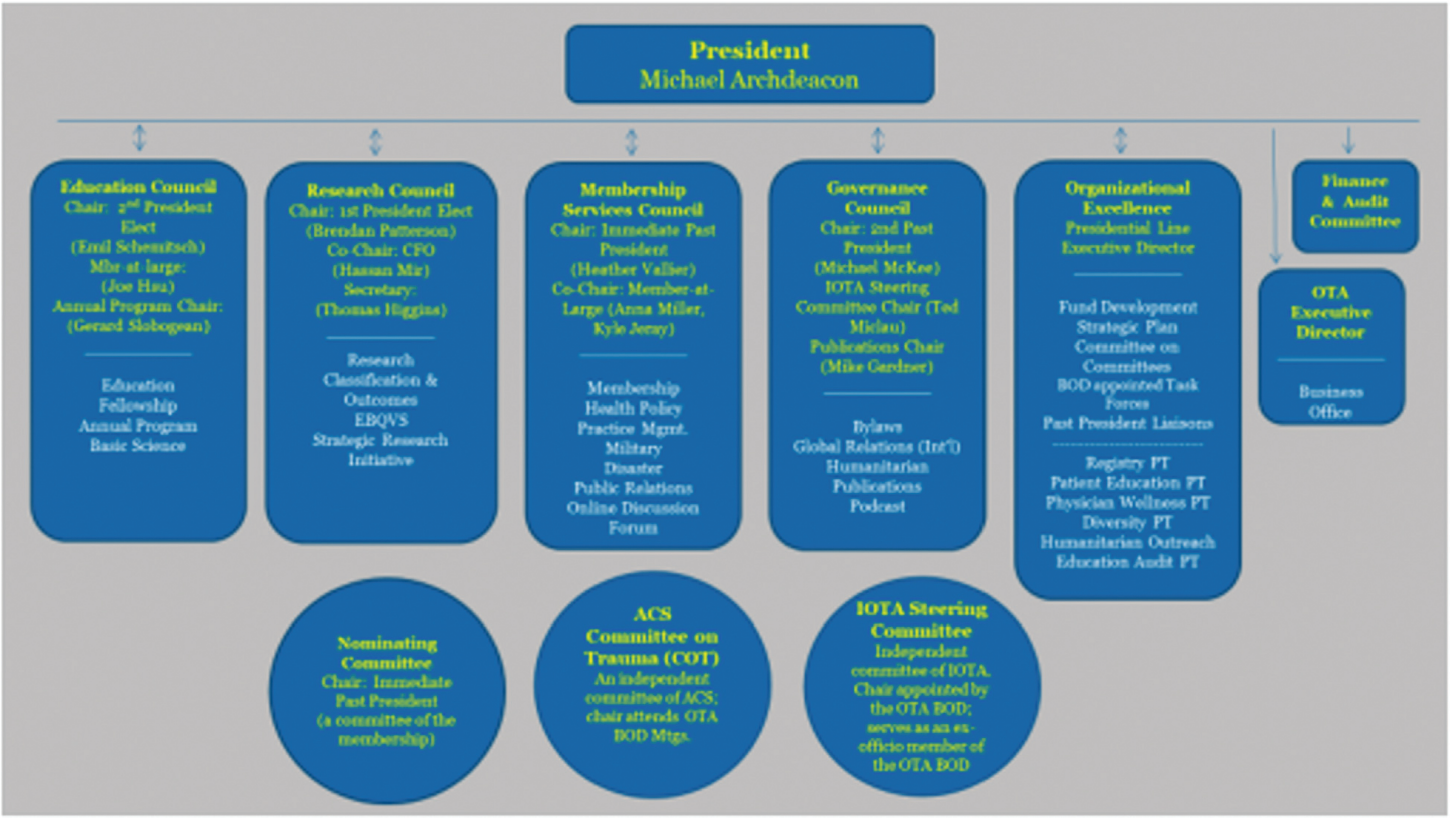
Orthopaedic Trauma Association board.

All these changes come along with changes in the OTA governance structure that facilitate more direct involvement and representation of the committees that deal with Intl. members or members – to – be (Humanitarian committee, Intl. Committee, Global Relations committee) (Fig. 1), a change that has been decided just recently by the OTA board (Fig. 2).

This special supplement to the OTA International journal is a representation of this growing body of global outreach. It was organized by members of the Intl. Committee and dedicated surgeons associated with the OTA Guest Nation program, the IOTA and the OTA Humanitarian committee. It will hopefully represent another piece in the puzzle of growing international exchange and growth of the ideas of the OTA.

